# DEVELOPMENT OF A RISK SCREEN OF ELDER ABUSE IN CAREGIVERS OF PEOPLE LIVING WITH DEMENTIA

**DOI:** 10.1093/geroni/igae098.2053

**Published:** 2024-12-31

**Authors:** Bonnie Olsen, Laura Mosqueda

**Affiliations:** University of Southern California, Los Angeles, California, United States; University of Southern California Keck School of Medicine, Pasadena, California, United States

## Abstract

Significant advances have been made in understanding risk factors of abuse of persons living with dementia (PLWD) by their caregivers. Intervention focused on the modifiable caregiver risk factors provides a possible avenue to primary prevention. This project developed a caregiver risk assessment screen, linked to an abuse prevention intervention. To develop the tool, we administered surveys of validated measures of risk factors to caregivers of PLWD (N=95), had an expert panel rank responses according to risk, and used these risk levels to identify items that are most predictive of risk. The sample had a mean age of 49.5 years (SD=14.4), mostly female (68.1%), mostly Latine (52.1%), predominantly family members (14.7% spouse, 43.1% child, 20.0% other). The PLWD had a mean age of 78.6 years (SD=10.8), mostly female (62.4%). Expert panel-assigned risk levels were evenly distributed, with 35.8% high, 30.5% medium, and 33.7% low risk. Using a 2:1 train/test split, the training sample demonstrated strong single-item prediction, with maximum single-item AUC (area under the curve) of 0.861 (39 items had AUC≥0.7, of which 5 had AUC≥0.8). The top performing items included: caregiver’s satisfaction with their life; acting in an emotionally aggressive manner; feeling angry when around the PLWD; being bothered by feeling nervous, anxious, or on edge; and finding looking after the PLWD to be a burden. This presentation will discuss the process of creating an abuse screening tool for caregivers of PLWD. In the next phase, we will assess the effectiveness of our intervention in reducing risk for abuse.

